# Synthesis and Pharmacological Studies of Unprecedented Fused Pyridazino[3′,4′:5,6][1,2,4]triazino[3,4-*b*][1,3,4]thiadiazine Derivatives

**DOI:** 10.3390/molecules23051024

**Published:** 2018-04-27

**Authors:** Rania S. Ali, Hosam A. Saad

**Affiliations:** 1 Department of Chemistry, Faculty of Science, Taif University, Taif 21974, Saudi Arabia; h_saadzag@yahoo.com; 2 Department of Basic Science, Faculty of Industrial Education, Helwan University, Cairo 11795, Egypt; 3 Department of Chemistry, Faculty of Science, Zagazig University, Zagazig 44511, Egypt

**Keywords:** pyridazino[3′,4′:5,6][1,2,4]triazino[4,3-*b*][1,2,4,5]tetrazine, antitumor activity, biological applications, cyclization reactions

## Abstract

A novel fused system with three or four fused rings—pyridazino[3′,4′:5,6][1,2,4]triazino[4,3-*b*][1,2,4,5]tetrazine and pyridazino[3′,4′:5,6][1,2,4]triazino[3,4-*b*]pyrimido[4,5-*e*][1,3,4]thiadiazine was obtained from the starting materials 4(6*H*)-amino-3-hydrazino-7-(2-thienyl)pyridazino[3,4-*e*][1,2,4]-triazine **2** and 9-amino-3-(2-thienyl)-2*H*,8*H*-pyridazino[3′,4′:5,6][1,2,4]triazino[3,4-*b*][1,3,4]thiadiazine-8-carbonitrile **12**. Each of the starting compounds was subjected to a number of cyclization reactions to obtain a series of new heterocyclic fused systems, **3**–**10** and **13**–**23**, via bifunctional reagents. Some of the synthesized compounds were screened against three cell lines including HepG2, HCT-116 and MCF-7 to discover their anticancer activity. The synthesized compounds were characterized depending on their elemental analyses and spectral data.

## 1. Introduction

As the world’s population increases, so too does the number of health problems, and the need to discover new therapeutics becomes even more urgent. Drug design represents the greatest hope for success in the present and future era. Discovering new drugs means saving the lives of many people. Heterocyclic compounds are widely distributed everywhere and area mainstay for life. Now, a vast number of heterocyclic compounds are pharmacologically active and are actually in clinical use [1]. To make scientific progress in the field of drug discovery, our main goal was to discover new drug classes with much better therapeutic profiles. In this paper, we synthesized two novel heterocyclic fused systems containing mainly thiophene as a substituent in pyridazinotriazine, which fused with tetrazine, thiadiazine, pyridine, and pyrimidine, rings in one way or another.

Thiophene and its derivatives have been widely distributed in many naturally occurring compounds and are employed to address different health hazards. They are responsible for various biological activities such as anti-inflammatory [2], antipyretic [3], anti-hypotensive [4], anti-convulsant [5], anti-viral [6], antitumor [7], fungicidal [8], herbicidal [9], anti-microbial [10] activities, and act as a plant-growth regulator [11]. Some of the thiophene derivatives exhibited divergences in anti-diabetic and anti-inflammatory activities [12].

Among a wide variety of 1,2,4-triazines screened for anticancer activities, a variety of fused 1,2,4-triazines have been reported to be extremely potent. Several heterobicyclic systems containing the 1,2,4-triazine ring have shown important biological activities. The imidazo[2,1-*c*][1,2,4]triazin-4(6*H*)-one core system showed noticeably lower cytotoxicity towards normal cells and several-times higher cytotoxicityagainst cancer cell lines [13,14,15,16,17]. Additionally, numerous heterobicyclic systems bearing the 1,2,4-triazine moiety possess lung, leukemia, CNS, and breast anticancer activities [18,19,20]. Pyrazolo[5,1-*c*][1,2,4]benzotriazine derivatives showed selective cytotoxicity on the human colorectal adenocarcinoma cell line HCT-8 in hypoxic conditions as well as normoxic conditions [21,22]. Several fused 1,2,4-trizines showed inhibitory activities on CYP1A1 [23].

In addition, a 1,2,4-triazine ring condensed with pyrimidine acts as an inhibitor of chaperone Heat-shock protein 90 (Hsp90) and, therefore, a potential anticancer agent [24]. Among fused 1,2,4-triazines, 8-(2-methoxyphenyl)-3,4-dioxo-6-thioxo-3,4,6,7-tetrahydro-2*H*-pyrimido[6,1-*c*][1,2,4] triazine-9-carbonitrile induced a significant growth inhibition of liver cancer cells HEPG2, which resulted to be equally as powerful as Doxorubicin and more potent than 5-fluorouracil used as reference drugs [25,26]. Other pyridotriazines were described as inhibitors of cyclin-dependent kinase or tyrosine kinase enzymes [27]. Substituted pyrimido[5,4-*e*][1,2,4]triazine-5,7(1*H*,6*H*)diones and pyrimido[5,4-*e*][1,2,4]triazine-5,7(6*H*,8*H*)diones were found active in antagonizing the b-catenin/TCF complex [28], which represents a useful tool for the treatment of cell proliferative disorders such as colorectal cancers. The 3-(4-(2-(Diethylamino)ethoxy)phenyl)-1,6-dimethylpyrimido[5,4-*e*][1,2,4]triazine-5,7(1*H*,6*H*)-dione showed the highest activity with an IC_50_ of 0.016 mM, the best LD_50_/IC_50_ ratio (8.32), and a favorable pharmacokinetic profile [28]. Recently, compound 2-(*p*-nitrobenzylidene)-4-phenyl-thioxo-triazino[2,1-*a*]-1,2,4-triazine-1,7-dione showed high activity against the HepG-2 cell line, showing an IC_50_ of 2.67 mM [29].

## 2. Results and Discussion

Once in a while, we look for unprecedented fused systems. Our challenge this time is to use the previously prepared compounds **1** and **11** [30,31] as a kernel for constructing a new fused system through subsequent reactions with a bifunctional reagent.

**Scheme 1:**
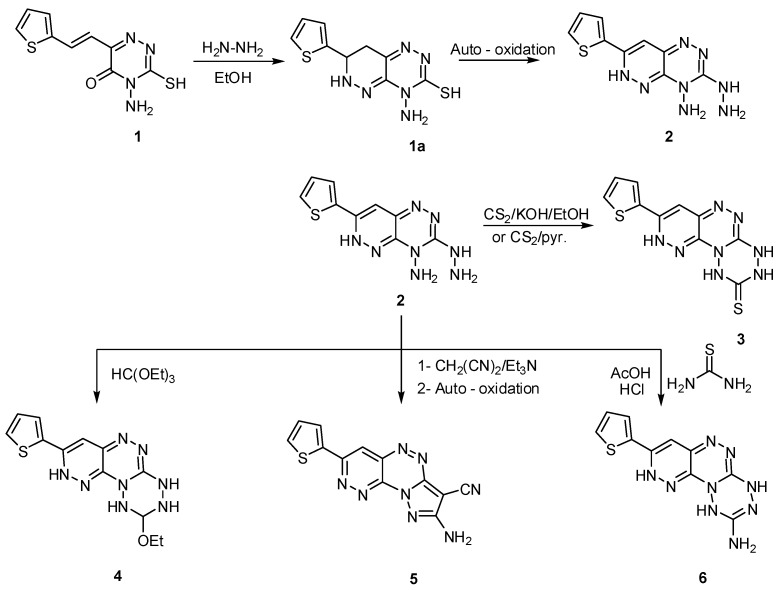
Synthesis and some reactions on 3-hydrazinyl-7-(thiophen-2-yl)pyridazino[3,4-*e*][1,2,4]triazin-4(6*H*)-amine.

The simple molecule 4-amino-3-mercapto-6-[2-(2-thienyl)vinyl]-1,2,4-triazin-5(4*H*)-one **1** reacted with hydrazine hydrate in refluxed ethanol to give the starting compound 4(6*H*)-amino-3-hydrazino-7-(2-thienyl)pyridazino[3,4-*e*][1,2,4]triazine **2**. This compound showed in its ^1^H-NMR the disappearance of each SH group and the two olefinic protons of the side chain double bond in compound **1** and its IR supported the observations where the amide carbonyl also disappeared. These observations led to the conclusion that the hydrazine hydrate cyclizes the side chain double bond with the carbonyl group to give the pyridazine ring along with the substitution of the SH group with the hydrazine group (Scheme 1). Compound **2** reacted with carbon disulphide either on alcoholic KOH and/or pyridine to give the new target heterocylic fused system known as pyridazino[3′,4′:5,6][1,2,4]triazino[4,3-*b*][1,2,4,5]tetrazine-9(10*H*)-thione derivative **3**. The same was obtained when compound **2** reacted with triethyl orthoformate, thiourea, formamide, acetic formic anhydride, and sebacoyl chloride. Reaction of **2** with triethyl orthoformate in boiling DMF gave 9-ethoxy-3-(2-thienyl)-7,8,9,10-tetrahydro-2*H*-pyridazino[3′,4′:5,6][1,2,4]triazino[4,3-*b*][1,2,4,5]tetrazine **4**. In addition, the reaction of compound **2** with malononitrile in basic medium yielded 8-Amino-3-(2-thienyl)pyrazolo[5,1-*c*]pyridazino[3,4-*e*][1,2,4]triazine-7-carbonitrile **5** through the nucleophilic substitution of the hydrazino group followed by cyclization and in situ auto-oxidation for aromatization of the diazole ring; reaction with thiourea in boiling acetic acid yielded 9-amino-3-(2-thienyl)-7,10-dihydro-2*H*-pyridazino[3′,4′:5,6][1,2,4]triazino[4,3-*b*][1,2,4,5]tetrazine **6** (Scheme 1). The structure’s characterization of the yielded compounds was based on their spectral data and the elemental analysis. Compound **3** in its mass spectra molecular ion peak M^+^ at 304 (22.1) and its IR showed a + signals due to the formation of a cyanodiazole ring. On the other hand, the structure of 9-aminotetrazino derivative **6** was confirmed from its ^1^H-NMR where it showed a signal due to NH_2_ and 3 NH groups at δ = 5.68, 9.82, 11.37 and 13.05 ppm, respectively.

**Scheme 2:**
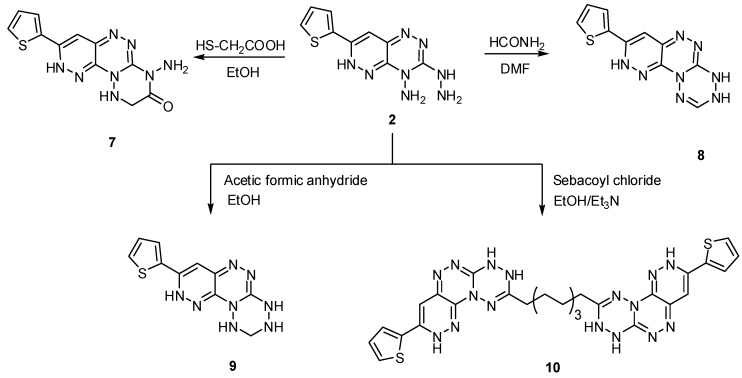
Some reactions on 4(6*H*)-amino-3-hydrazinyl-7-(thiophen-2-yl)pyridazino[3,4-*e*][1,2,4]triazine.

The reaction of compound **2** with thioglycolic acid in boiling ethanol yielded 7-amino-3-(2-thienyl)-2,7,9,10-tetrahydro-8*H*-pyridazino[3,4-*e*][1,2,4]triazino[3,2-*c*][1,2,4]triazin-8-one **7** while, with formamide in refluxing, DMF gave 3-(2-thienyl)-7,8-dihydro-2*H*-pyridazino[3′,4′:5,6][1,2,4]triazino[4,3-*b*][1,2,4,5]tetrazine **8** (Scheme 2). The structures of compounds **7** and **8** were confirmed from their ^1^H-NMR and IR where the IR of compound **7** showed a band due to an amide carbonyl at 1654 cm^1^ and no evidence supported the presence of S atom functional groups such as SH or C=S and this means that the reaction was carried out through the nucleophilic substitution on thioglycolic acid CH_2_ followed by cyclization to give the triazinone ring. The ^1^H-NMR supported this elucidation by the appearance of doublets due to the electronic environment unsymmetrical CH_2_ signal at δ = 3.65, 3.89 ppm. The ^1^H-NMR of compound **8** showed no NH_2_ group signals and its ^13^C-NMR showed an extra signal due to a carbon atom for the formed tetrazine ring.

The 3-(2-thienyl)-7,8,9,10-tetrahydro-2*H*-pyridazino[3′,4′:5,6][1,2,4]triazino[4,3-*b*][1,2,4,5]-tetrazine **9** was prepared by the reaction of compound **2** with acetic formic anhydride [32] in boiling ethanol while the dimmer **10** was formed from the reaction of 2 with sebacoyl chloride in basic conditions (Scheme 2). The structures of compounds 9 and 10 confirmed from their spectral date where the two compounds showed the disappearance of the two NH_2_ groups of compound **2** in both IR and ^1^H-NMR. The ^13^C-NMR supported this conclusion by showing signals due to sp^3^ carbons in the range δ = 23.68–55.29 ppm.

**Scheme 3:**
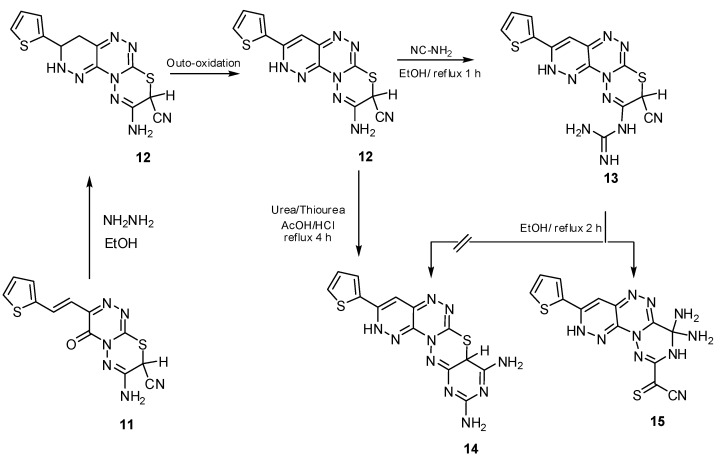
Synthesis and some reactions of 3-amino-3-(2-thienyl)-2*H*,8*H*-pyridazino[3′,4′:5,6][1,2,4]triazino[3,4-*b*][1,3,4]thiadiazine-8-carbonitrile.

**Scheme 4:**
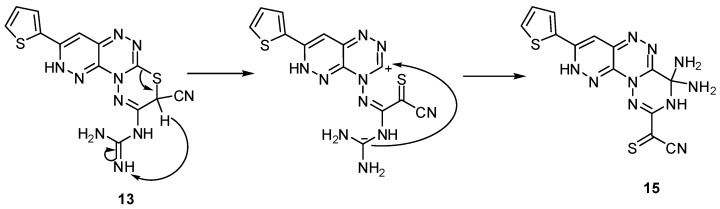
The mechanism of formation of compound **15**.

Compound **12** was prepared by reaction of compound **11** [31] with hydrazine hydrate to give the new hetrocylic system pyridazino[3′,4′:5,6][1,2,4]triazino[3,4-*b*][1,3,4]-thiadiazine, which is an unprecedented system. Compound **12** acts as a starting material in some cyclization reactions where it reacted with cyanamine in an EtOH/H_2_O mixture (4:1 by volume) to give the guanidine derivative **13**. The idea of cyclization of compound **13** was intuitive from the appearance of two spots in TLC during compound **13** synthesis. Therefore, we thought to repeat the synthesis of compound **13** but with a longer time reflux. This reaction resulted in [7,7-diamino-3-(2-thienyl)-7,8-dihydro-2*H*-pyridazino-[3,4-*e*][1,2,4]triazino[6,1-*c*][1,2,4]triazin-9-yl](thioxo)acetonitrile **15**. Compound **14** was formed by treating compound **12** with urea and/or thiourea in boiling acetic acid (Scheme 3). Compound **12** showed in its IR the disappearance of the amide carbonyl to confirm the formation of the pyridazine ring. The structure of compound **13** was recognized from its ^1^H-NMR where it showed three NH signals instead of one in compound **12**. The structure of compound **14** was proven by the disappearance of the CN group in its IR chart with the appearance of another NH_2_ in both IR and ^1^H-NMR. The mechanism describes the cyclization of compound **13** shown in Scheme 4 [31]. The structure of compound **15** showed an absorption band at 1345 cm^−1^ due to C=S with other bands at 3376–3212 cm^−1^ and 2207 cm^−1^ due to the 2NH_2_ and CN groups, respectively.

**Scheme 5:**
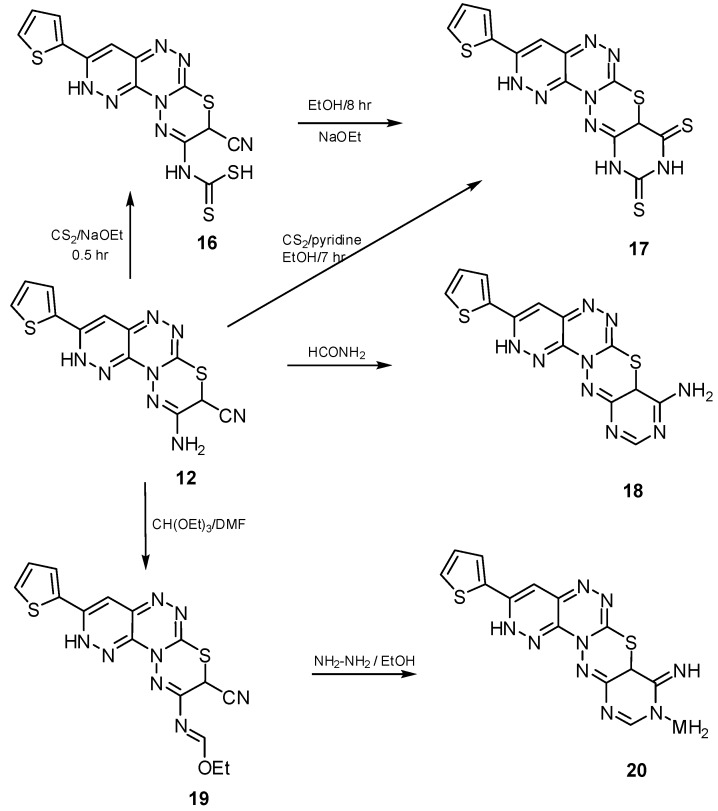
Some reactions of 3-amino-3-(2-thienyl)-2*H*,8*H*-pyridazino[3′,4′:5,6][1,2,4]triazino[3,4-*b*][1,3,4]thiadiazine-8-carbonitrile.

The [8-cyano-3-(2-thienyl)-2*H*,8*H*-pyridazino[3′,4′:5,6][1,2,4]triazino[3,4-*b*][1,3,4]thiadiazin-9-yl]carbamodithioic acid **16**, formed from the reaction of compound **12** with carbon disulphide in alcoholic KOH, was cyclized to 3-(2-thienyl)-2*H*,7a*H*-pyridazino[3′,4′:5,6][1,2,4]triazino[3,4-*b*]pyrimido[4,5-*e*]-[1,3,4]thiadiazine-8,10(9*H*,11*H*)dithione **17** either by the long boiling for compound **16** in ethanol or by direct reaction of compound 12 with CS_2_ in pyridine (Scheme 5).

Formamide with compound **12** in boiling DMF gave 8-amino-3-(2-Thienyl)-2*H*,7a*H*-pyridazino[3′,4′:5,6][1,2,4]triazino[3,4-*b*]pyrimido-[4,5-*e*][1,3,4]thiadiazine **18** while with triethyl orthoformate in DMF it yielded ethyl [8-cyano-3-(2-thienyl)-2*H*,8*H*-pyridazino[3′,4′:5,6][1,2,4]triazino[3,4-*b*][1,3,4]-thiadiazin-9-yl]imidoformate **19**, which on reaction with hydrazine hydrate in boiling EtOH gave 9-amino-8-imino-3-(2-thienyl)-7a,8-dihydro-2*H*,9*H*-pyridazino[3′,4′:5,6][1,2,4]-triazino[3,4-*b*]pyrimido[4,5-*e*]-[1,3,4]thiadiazine **20** (Scheme 5).

To confirm the above structures, we scrutinized their IR, ^1^H-NMR, and ^13^C-NMR. The IR of compound **16** showed a new band at 2613 and 1345 cm^−1^ due to SH and C=S groups with the disappearance of the NH_2_ group of compound **12**.Additionally, its ^1^H-NMR showed the most important signal at δ = 13.78 ppm, corresponding to the SH group. In the case of compound **17**, the groups that underpin the structure of compound **16** vanished and new groups characteristic of the compound **17** structure appeared. The ^1^H-NMR of **17** showed the demise of the SH group and a third NH group appeared. The IR of compound **18** showed the disappearance of the CN group, which contributes to the cyclization process. The ^1^H-NMR of **19** illustrates a doublet and triplet at δ = 1.20 and 3.33 due to CH_3_ and CH_2_, respectively. Additionally, ^13^C-NMR supported this evidence; it showed signals due to CH_3_ and CH_2_ at δ = 13.7 and 62.59 ppm, which disappeared in compound **20** ^1^H-NMR.

**Scheme 6:**
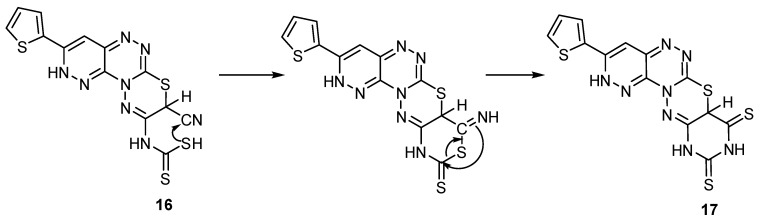
Mechanism of cyclization of compound **16.**

The postulated mechanism illustrates the cyclization of compound **17** shown in Scheme 6.

**Scheme 7:**
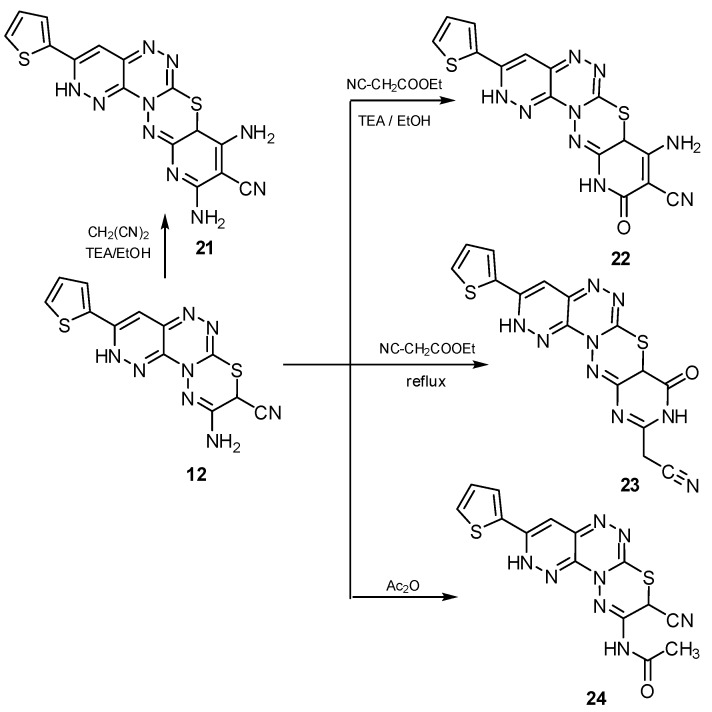
Some reactions of 9-amino-3-(2-thienyl)-2*H*,8*H*-pyridazino[3′,4′:5,6][1,2,4]triazino[3,4-*b*][1,3,4]thiadiazine-8-carbonitrile.

The 8,10-diamino-3-(2-thienyl)-2*H*,7a*H*-pyridazino[3′,4′:5,6][1,2,4]triazino[3,4-*b*]pyrido[2,3-*e*][1,3,4]thiadiazine-9-carbonitrile **21** resulted from the reaction of compound **12** with malononitrile in basic conditions. The reaction of compound **12** with ethyl cycnoacetate yielded two different compounds, **22** and **23**, according to the condition of the reaction. In addition, boiling of compound **12** with acetic anhydride yielded *N*-[8-cyano-3-(2-thienyl)-2*H*,8*H*-pyridazino[3′,4′:5,6][1,2,4]triazino[3,4-*b*][1,3,4]-thiadiazin-9-yl]acetamide **24 (**Scheme 7). The structure of compound **21** was elucidated from ^1^H-NMR to ^13^C-NMR where the ^1^H-NMR showed two amino groups at δ = 6.02 and 6.59 ppm while ^13^C-NMR showed an extra three carbons more than compound **12** due to the pyridine ring formed.

**Scheme 8:**
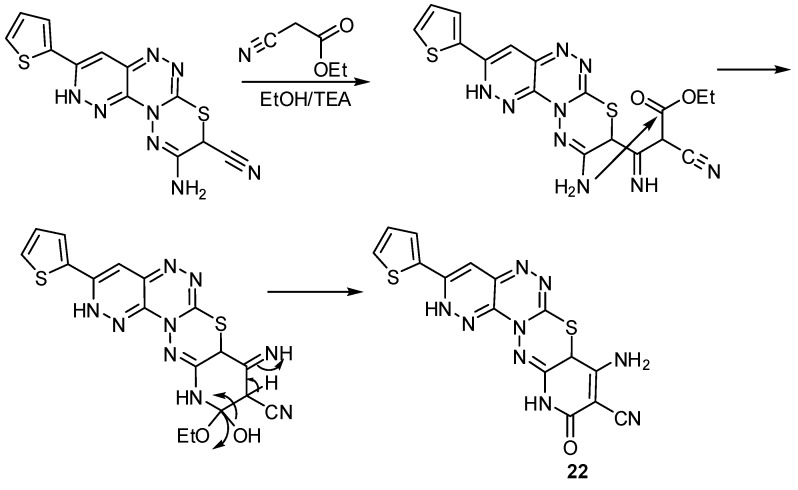
Mechanism of formation of compound **22**.

**Scheme 9:**
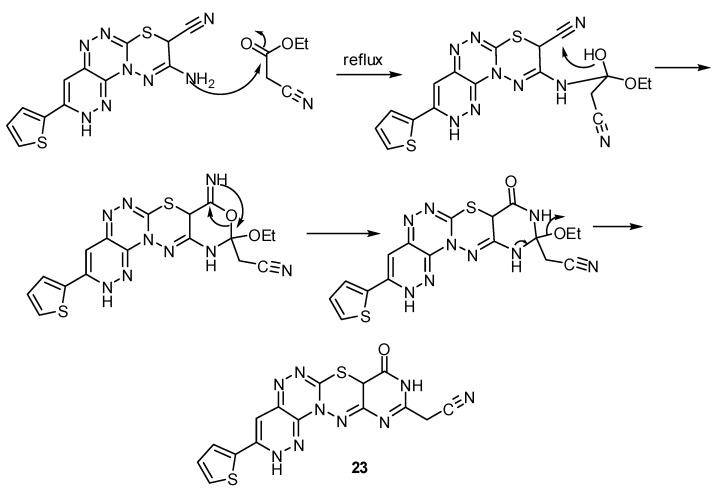
Mechanism of formation of compound **23**.

The structure formed from the reaction of ethyl cyanoacetate in basic conditions, 8-amino-10-oxo-3-(2-thienyl)-10,11-dihydro-2*H*,7a*H*-pyridazino[3′,4′:5,6][1,2,4]-triazino[3,4-*b*]pyrido[2,3-*e*][1,3,4]thiadiazine-9-carbonitrile **22**, was confirmed from the compound IR where it showed an amide carbonyl and its ^1^H-NMR showed no methylene group like that found in compound **23**, which resulted from the reaction of compound **12** with boiling ethyl cyanoacetate. The mechanisms describe the formation of compounds **22** and **23**, which are shown in Scheme 8 and Scheme 9, respectively.

### Cytotoxic Activity

**Figure 1:**
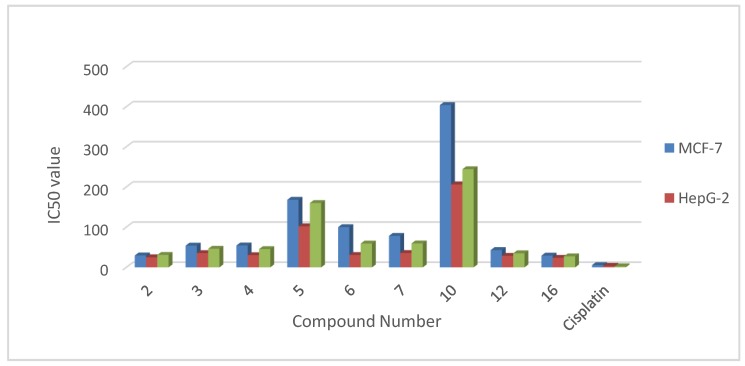
The anti-tumor activities of the tested compounds expressed as IC_50_ values and compared with reference standard drugs evaluated on breast cancer, liver cancer, and colon carcinoma cell lines.

The in vitro growth inhibitory activity of the synthesized compounds was investigated in comparison with the well-known anticancer standard drugs (cisplatin) under the same conditions using colorimetric MTT assay. Data generated were used to plot a dose–response curve of which the concentration of test compounds required to kill 50% of cell population (IC_50_) was determined (see Figure 1 and Table 1). The results revealed that all the tested compounds showed inhibitory activity with the tumor cell lines in a concentration-dependent manner. Cytotoxic activity was expressed as the mean IC_50_ of three independent experiments.

The order of activity against the breast carcinoma cell line (MCF-7) was 16, 2, 12, 3, 4, 7, 6, 5 and 10, with IC_50_ values of 29.2 ± 0.7, 29.6 ± 0.9, 43.9 ± 1.2, 54 ± 0.8, 54.5 ± 1.2, 78.2 ± 1.2, 100 ± 0.9, 145 ± 3.4, 168 ± 4.5 and 404 ± 3.4 μg/mL, respectively.

The order of activity against the liver carcinoma cell line (HepG2) was 16, 2, 12, 4, 6, 7, 3, 5 and 10 with IC_50_ values of 23.6 ± 0.6, 25 ± 0.4, 28.6 ± 0.9, 30.2 ± 0.4, 30.7 ± 0.7, 35.6 ± 0.9, 35.5 ± 1.2, 98.1 ± 0.7, 102 ± 0.9 and 206 ± 2.3 μg/mL, respectively.

The order of activity against the colon carcinoma cell line (HCT-116) was 16, 2, 12, 4, 3, 6, 7, 5 and 10 with IC_50_ values of 27.4 ± 0.6, 30.9 ± 0.4, 34.9 ± 0.6, 45.4 ± 0.8, 46.2 ± 0.7, 59.3 ± 0.8, 59.5 ± 1.2, 160 ± 2.3, 198 ± 2.4 and 244 ± 3.9 μg/mL, respectively.

In conclusion, the results showed that compounds **16**, **2** and **12** were the most active against the three tested carcinoma cell lines (HepG2, MCF-7 and HCT-116) compared with cisplatin reference drugs.

Moreover, the compounds **5** and **10** were relatively less active against the tested tumor cell line.

## 3. Materials and Methods

### 3.1. General Information

All chemicals were purchased from Sigma (New York, NY, USA). The melting points were measured by a digital Electro thermal IA 9100 Series and were uncorrected. IR spectra were recorded on an ATRAlpha FTIR spectrophotometer (Billerica, MA, USA) from 400 cm^−1^ to 4000 cm^−1^. ^1^H-NMR and ^13^C-NMR spectra were recorded on a Brüker AC-600 MHz instrument (Bruker, Billerica, Massachusetts). Chemical shifts were expressed as ppm relative to TMS as an internal standard and DMSO-*d*_6_ was used as the solvent. Mass spectra were recorded on a Shimadzu GC-MS-QP 1000 EX spectrometer (Shimadzu, Kyoto, Japan). The pharmacological study was carried out at Al-Azhar University (Cairo, Egypt), The Regional Center for Mycology and Biotechnology. Elemental analyses were performed at the Micro-analytical Center, Cairo University (Cairo, Egypt).

*4(6H)-amino-3-hydrazino-7-(2-thienyl)pyridazino[3,4-e][1,2,4]triazine***2**. Hydrazine hydrate (5 mL, excess) was added to a solution of compound **1** [1] (2.52 g, 0.01 mol) in ethanol (20 mL) and the reaction mixture was refluxed for 9 h. The pale yellow precipitate formed after cooling was filtered and crystallized from ethanol to give pale yellow crystals of compound **2**. Yield, 89%, m.p.: 284–286 °C. IR: 3371–3193 cm^−1^ (2NH_2_ and 2NH) and 1621 cm^−1^ (C=N). ^1^H-NMR (DMSO-*d*_6_): δ = 5.52 (s, 1H, pyridazine CH), 5.57 (s, 2H, N-NH_2_), 6.57 (s, 2H, NH-NH_2_), 7.05 (dd appears t, 1H, *J* = 7.8, 7.2 Hz, thiophene-H*_C4_*), 7.26 (d, 1H, *J* = 7.8 Hz, thiophene-H*_C_*_3_), 7.58 (d, 1H, *J* = 10.2 Hz, thiophene-H*_C_*_5_), 7.92 (s, 1H, NH), 8.06 (s, 1H, NH). ^13^C-NMR (DMSO-*d*_6_): δ = 112.5, 118, 120.6, 123.4, 124.6, 141.1, 143.3, 148.0 and 151.8 (Ar-C, C=C, C-S and C=N). Anal. Calcd. for C_9_H_10_N_8_S (262.29): C, 41.21; H, 3.84; N, 42.72; S, 12.22; Found: C, 41.03; H, 3.71; N, 42.59; S, 12.10. LCMS (ESI) *m*/*z* (int. %) (263 (2.5)) [M + H]^+^; Calcd. for (262.29): 262 (11.1), 250 (48.03), 249 (100), 234 (12.68), 219 (12.27), 136 (20.39), 135 (38.11), 121 (11.76), 77 (10.93), 69 (11.81).

*3-(2-Thienyl)-7,8-dihydro-2H-pyridazino[3′,4′:5,6][1,2,4]triazino[4,3-b][1,2,4,5]tetrazine-9(10H)-thione***3**. Method A: A mixture of compound **2** (0.25 g, 0.001 mol) and carbon disulfide (1.5 mL) in ethanolic sodium ethoxide (20 mL, 0.07 g, 0.001 mol) was refluxed for 9 h. The mixture was poured onto ice water after cooling and acidified with HCl. The yellow solid formed was filtered and crystallized from DMF to give compound **3**. Yield, 68%, m.p.: over 300 °C. Method B: To a solution of **2** (0.25 g, 0.001 mol) in dry pyridine (15 mL), carbon disulfide (2 mL) was added and the mixture was refluxed for 12 h. After cooling, the solution was poured onto ice water and acidified with HCl. The obtained solid was separated by filtration and crystallized from DMF to give **3**. Yield 54%, m.p. over 300 °C. IR: 3271–3232 cm^−1^ (3NH), 1618 cm^−1^ (C=N) and 1348 cm^−1^ (C=S). ^1^H-NMR (DMSO-*d*_6_): δ = 6.70 (s, 1H, pyridazine CH), 7.09 (dd appears t, 1H, *J* = 9.6, 7.2 Hz, thiophene-H*_C_*_4_), 7.30 (d, 1H, *J* = 9.6 Hz, thiophene-H*_C_*_3_), 7.51 (d, 1H, *J* = 10.2 Hz, thiophene-H*_C_*_5_), 9.02 (s, 1H, NH), 11.95 (s, 1H, NH), 12.33 (s, 1H, NH), 12.62 (s, 1H, NH).^13^C-NMR (DMSO-*d*_6_): δ = 111.9, 118.0, 121.3, 127.0, 129.6, 131.6, 136.0, 140.2, 142.6 and 164.0 (Ar-C, C=C, C-S, C=N and C=S). Anal. Calcd. for C_10_H_8_N_8_S_2_(304.35): C, 39.46; H, 2.65; N, 36.82; S, 21.07; Found: C, 39.41; H, 2.55; N, 36.71; S, 20.95 LCMS (ESI) *m*/*z* (int. %) (305 (0.53)) [M + H]^+^; Calcd. For (304.35): 304 (22.1), 252 (47.06), 251 (44.27), 236 (20.22), 145 (10.20), 136 (18.00), 135 (21.15), 134 (12.64), 121 (28.22), 111 (17.14), 109 (20.35), 98 (19.05), 97 (100), 96 (19.95), 95 (19.11), 85 (29.47), 84 (25.05), 83 (39.25), 71 (50.87), 69 (59.75), 60 (28.03).

*9-Ethoxy-3-(2-thienyl)-7,8,9,10-tetrahydro-2H-pyridazino[3′,4′:5,6][1,2,4]triazino-[4,3-b][1,2,4,5]tetrazine***4**. A mixture of compound **2** (0.25 g, 0.001 mol) and triethyl orthoformate (3 mL) in DMF (10 mL) was refluxed for 4 h. The mixture was poured onto ice/water (40 mL).The solid, therefore, separated and was filtered. Then, it was crystallized from ethanol to give **4** as orange powder. Yield, (62%), m.p.: 269–271 °C. IR: 3269–3192 cm^−1^ (4NH), 1625 cm^−1^ (C=N). ^1^H-NMR (DMSO-*d*_6_): δ = 1.21 (t, 3H, *J* = 7.8, CH_3_), 3.41 (q, 2H, *J* = 12.8, CH_2_), 6.63 (s, 1H, pyridazine CH), 7.10 (dd appears t, 1H, *J* = 9.6, 7.2 Hz, thiophene-H*_C_*_4_), 7.33 (d, 1H, *J* = 9.6 Hz, thiophene-H*_C_*_3_), 7.57 (d, 1H, *J* = 10.2 Hz, thiophene-H*_C_*_5_), 9.14 (s, 1H, NH), 11.02 (s, 1H, NH), 11.21 (s, 1H, NH), 12.02 (s, 1H, NH).^13^C-NMR (DMSO-*d*_6_): δ = 25.18, 32.53 (CH_3_ and CH_2_), 116.8, 118.2, 123.5, 128.6, 129.0, 131.5, 138.5, 141.4, 151.4 and 159.2. (Ar-C, C=C, C-O, C-S and C=N). Anal. Calcd. for C_12_H_14_N_8_OS(318.36): C, 45.27; H, 4.43; N, 35.20; S, 10.07; Found: C, 45.22; H, 4.32; N, 35.05; S, 9.89. LCMS (ESI) *m*/*z* (int. %) (319 (0.15)) [M + H]^+^; Calcd. For (318.35): 318 (0.22), 260 (32.53), 252 (9.59), 162 (11.68), 145 (14.28), 136 (30.06), 135 (100), 121 (36.05), 109 (20.45), 108 (20.59), 97 (42.65), 91 (18.17), 69 (36.00), 63 (16.30), 60 (22.45).

*8-Amino-3-(2-thienyl)pyrazolo[5,1-c]pyridazino[3,4-e][1,2,4]triazine-7-carbonitrile***5**. Malononitrile (0.06 g, 0.001 mol) and triethylamine were added to a solution of compound **2** (0.25 g, 0.001 mol) in ethanol (20 mL) and the mixture was refluxed for 8 h. After cooling, the orange precipitate formed was filtered and crystallized from dioxane to give compound **5**. Yield, 63%, m.p.: over 300 °C. IR: 3382 cm^−1^ (NH_2_), 2210 cm^−1^ (CN), 162,219 cm^−1^ (C=N). ^1^H-NMR (DMSO-*d*_6_): δ = 6.51 (s, 2H, NH_2_), 7.05 (dd appears t, 1H, *J* = 7.2, 7.2 Hz, thiophene-H*_C_*_4_), 7.39 (d, 1H, *J* = 7.6 Hz, thiophene-H*_C_*_3_), 7.47 (d, 1H, *J* = 10.2 Hz, thiophene-H*_C_*_5_). ^13^C-NMR (DMSO-*d*_6_): δ = 106.8 (CN), 116.8, 120.4, 122.5, 128.6, 129.9, 133.2, 139.4, 144.1, 151.2, 153.4 and 158.3. (Ar-C, C=C, C-S and C=N). Anal. Calcd. for C_12_H_6_N_8_S(294.29): C, 48.97; H, 2.05; N, 38.08; S, 10.90; Found: C, 48.72; H, 1.91; N, 37.88; S, 10.68. LCMS (ESI) *m*/*z* (int. %) (295 (0.87)) [M + H]^+^; Calcd. For (294.29): 294 (1.57), 253 (14.84), 252 (69.11), 251 (72.55), 250 (47.80), 249 (100), 162 (14.83), 145 (13.48), 136 (33.17), 135 (54.51), 121 (2.98), 109 (14.47), 108 (8.53), 97 (26.33), 77 (17.76), 69 (19.04), 63 (9.66), 60 (8.58).

*9-Amino-3-(2-thienyl)-7,10-dihydro-2H-pyridazino[3′,4′:5,6][1,2,4]triazino[4,3-b][1,2,4,5]tetrazine***6**. A mixture of compound **2** (0.25 g, 0.001 mol) and thiourea (0.076 g, 0.001 mol) was refluxed in glacial acetic acid (10 mL) containing HCl (1 mL) as a catalyst for 3 h. After cooling, the mixture was poured onto ice-water and neutralized with ammonia solution. The solid, therefore, separated and was filtered and crystallized from methanol to give yellow crystals. Yield, 61% m.p.: over 300 °C. IR: 3382–3236 cm^−1^ (NH_2_, 3NH) and 1622 cm^−1^ (C=N). ^1^H-NMR (DMSO-*d*_6_): δ = 5.68 (s, 2H, NH_2_), 6.77 (s, 1H, pyridazine CH), 7.09 (dd appears t, 1H, *J* = 7.2, 7.2 Hz, thiophene-H*_C_*_4_), 7.33 (d, 1H, *J* = 7.6 Hz, thiophene-H*_C_*_3_), 7.48 (d, 1H, *J* = 10.2 Hz, thiophene-H*_C_*_5_), 9.82 (s, 1H, NH), 11.37 (s, 1H, NH), 13.05 (s, 1H, NH). ^13^C-NMR (DMSO-*d*_6_): δ = 118.2, 127.6, 128.3, 128.5, 129.4, 141.0, 141.1, 148.0, 152.3 and 158.3. (Ar-C, C=C, C-S and C=N). Anal. Calcd. for C_10_H_9_N_9_S(287.30): C, 41.80; H, 3.16; N, 43.88; S, 11.16; Found: C, 41.74; H, 3.09; N, 43.81; S, 11.03. LCMS (ESI) *m*/*z* (int. %) (288 (0.24)) [M + H]^+^; Calcd. For (287.30): 287 (0.39), 253 (20.26), 252 (89.92), 251 (100), 193 (10.10), 162 (14.59), 145 (20.58), 136 (26.98), 135 (40.27), 143 (23.34), 121 (52.87), 109 (22.51), 108 (16.18), 97 (16.05), 77 (31.66), 69 (33.64), 63 (18.45), 60 (39.19).

*7-Amino-3-(2-thienyl)-2,7,9,10-tetrahydro-8H-pyridazino[3,4-e][1,2,4]triazino[3,2-c][1,2,4]triazin-8-one***7**. Thioglycolic acid (0.092 g, 0.07 mL, 0.001 mol) and compound **2** (0.25 g, 0.001 mol) in EtOH (15 mL) were refluxed for 6 h. After cooling, the solution was poured onto cold water. The solid formed was filtered and crystallized from ethanol to give yellowish orange crystals. Yield, 52% m.p.: over 300 °C. IR: 3366–3247 cm^−1^ (NH_2_, 2NH), 1654 cm^−1^ (C=O) and 1624 cm^−1^ (C=N). ^1^H-NMR (DMSO-*d*_6_): δ = 3.65, 3.89 (dd, 2H, CH_2_), 5.69 (s, 2H, NH_2_), 6.50 (s, 1H, pyridazine CH), 7.05 (dd appears t, 1H, *J* = 7.2, 7.2 Hz, thiophene-H*_C_*_4_), 7.31 (d, 1H, *J* = 7.6 Hz, thiophene-H*_C_*_3_), 7.47 (d, 1H, *J* = 10.2 Hz, thiophene-H*_C_*_5_), 9.65 (s, 1H, NH), 11.17 (s, 1H, NH).^13^C-NMR (DMSO-*d*_6_): δ = 118.9, 127.2, 128.4, 128.8, 129.0, 141.4, 141.8, 147.9, 151.7, 153.6 and 161.3. (Ar-C, C=C, C=O, C-S and C=N). Anal. Calcd. for C_11_H_10_N_8_OS(302.32): C, 43.70; H, 3.33; N, 37.07; S, 10.61; Found: C, 43.55; H, 3.21; N, 36.91; S, 10.52.

*3-(2-Thienyl)-7,8-dihydro-2H-pyridazino[3′,4′:5,6][1,2,4]triazino[4,3-b][1,2,4,5]tetrazine***8**. A mixture of **2** (0.25 g, 0.001 mol) and formamide (3 mL) in dimethylformamide (15 mL) was refluxed for 3 h. The reaction mixture cooled and was then poured onto cold water (30 mL). The solid formed was filtered and crystallized from DMF to give **8** as a brownish powder. Yield, (58%), m.p.: over 300 °C. IR: 3255–3185 cm^−1^ (3NH), 1618 cm^−1^ (C=N). ^1^H-NMR (DMSO-*d*_6_): δ = 6.58 (s, 1H, pyridazine CH), 7.05 (dd appears t, 1H, *J* = 7.2, 7.2 Hz, thiophene-H*_C_*_4_), 7.02 (s, 1H, tetrazine CH), 7.33 (d, 1H, *J* = 7.6 Hz, thiophene-H*_C_*_3_), 7.48 (d, 1H, *J* = 10.2 Hz, thiophene-H*_C_*_5_), 9.85 (s, 1H, NH), 11.34 (s, 1H, NH), 11.68 (s, 1H, NH). ^13^C-NMR (DMSO-*d*_6_): δ = 118.9, 127.2, 128.4, 128.8, 129.0, 141.4, 141.8, 147.9, 151.7, 153.6 and 161.3. (Ar-C, C=C, C=O, C-S and C=N). Anal. Calcd. for C_10_H_8_N_8_S (272.29): C, 44.11; H, 2.96; N, 41.15; S, 11.78; Found: C, 44.02; H, 2.88; N, 41.13; S, 11.68.

*3-(2-Thienyl)-7,8,9,10-tetrahydro-2H-pyridazino[3′,4′:5,6][1,2,4]triazino[4,3-b]-[1,2,4,5]tetrazine***9**. A mixture of acetic formic anhydride [32] and compound **2** (1:1, 0.001 mole) in ethanol were refluxed for 5 h. The mixture was poured onto ice water (35 mL). The precipitate formed crystallized from ethanol after filtration and drying to give compound **9** as a yellow powder. Yield, (58%), m.p.: 293–295 °C. IR: 3274–3191 cm^−1^ (4NH), 1625 cm^−1^ (C=N). ^1^H-NMR (DMSO-*d*_6_): δ = 3.65 (s, 2H, tetrazine CH_2_), 6.51 (s, 1H, pyridazine CH), 7.05 (dd appears t, 1H, *J* = 7.8, 7.2 Hz, thiophene-H*_C_*_4_), 7.30 (d, 1H, *J* = 6.6 Hz, thiophene-H*_C_*_3_), 7.45 (d, 1H, *J* = 9.6 Hz, thiophene-H*_C_*_5_), 9.85 (s, 1H, NH), 11.34 (s, 1H, NH), 11.61 (s, 1H, NH), 11.68 (s, 1H, NH). ^13^C-NMR (DMSO-*d*_6_): δ = 55.29 (CH_2_), 119.2, 123.2, 128.8, 129.5, 141.1, 148.8, 151.6, 155.6 and 156.1. (Ar-C, C=C, C-S and C=N). Anal. Calcd. for C_10_H_10_N_8_S(274.31): C, 43.79; H, 3.67; N, 40.85; S, 11.69; Found: C, 43.64; H, 3.59; N, 40.79; S, 11.61.

*9,9′-Octane-1,8-diylbis[3-(2-thienyl)-7,8-dihydro-2H-pyridazino[3′,4′:5,6][1,2,4]triazino[4,3-b][1,2,4,5]tetrazine***10**. A mixture of **2** (0.25 g, 0.001 mol) and sebacoyl chloride (0.24 g, 0.21 mL, 1 mmol) and TEA (catalytic amount) in DMF (15 mL) was refluxed for 4 h. The mixture was cooled and poured onto cold ice-water. The solid formed was filtered and then crystallized from ethanol to give compound **10** as yellow crystals. Yield 87%, m.p. 294–296 °C. IR: 3292–3264 cm^−1^ (3NH), 1624 cm^−1^ (C=N). ^1^H-NMR (DMSO-*d*_6_): δ = 1.35 (m, 4H, 2CH_2_), 1.54 (m, 4H, 2CH_2_), 1.60 (m, 4H, 2CH_2_), 2.67 (t, 4H, CH_2_), 3.65 (s, 2H, tetrazine CH_2_),5.92 (s, 2H, pyridazine CH), 7.03 (dd appears t, 2H, *J* = 7.8, 7.2 Hz, thiophene-H*_C_*_4_), 7.35 (d, 2H, *J* = 6.6 Hz, thiophene-H*_C_*_3_), 7.41 (d, 2H, *J* = 9.6 Hz, thiophene-H*_C_*_5_), 9.82 (s, 2H, NH), 11.30 (s, 2H, NH), 11.69 (s, 2H, NH). ^13^C-NMR (DMSO-*d*_6_): δ = 23.68, 25.12, 29.50, 31.22 (4 CH_2_), 110.2, 120.2, 125.3, 129.9, 147.9, 152.5, 155.6, 156.7, 158.3 and 158.9. (Ar-C, C=C, C-S and C=N). Anal. Calcd. for C_28_H_30_N_16_S_2_(654.77): C, 51.36; H, 4.62; N, 34.23; S, 9.79; Found: C, 51.19; H, 4.51; N, 34.09; S, 9.65.

*9-Amino-3-(2-thienyl)-2H,8H-pyridazino[3′,4′:5,6][1,2,4]triazino[3,4-b][1,3,4]thiadiazine-8-carbonitrile***12**. To a solution of compound **11** (3.16 g, 0.01 mol) in ethanol (25 mL), Hydrazine hydrate (10 mL, excess) was added and the reaction mixture was refluxed for 9 h. The pale yellow precipitate formed after cooling was filtered and crystallized from ethanol to give red crystals of compound **12**. Yield, 92%, m.p.: over 300 °C. IR: 3317–3109 cm^−1^ (NH_2_ and NH), 2208 cm^−1^ (C≡N) cm^−1^ and 1623 cm^−1^ (C=N). ^1^H-NMR (DMSO-*d*_6_): 3.63 (s, 1H, thiadiazine CH), 5.88 (s, 1H, pyridazine CH), 6.02 (s, 1H, NH_2_), 7.05 (dd appears t, 1H, *J* = 7.8, 7.2 Hz, thiophene-H*_C_*_4_), 7.33 (d, 1H, *J* = 6.6 Hz, thiophene-H*_C_*_3_), 7.43 (d, 1H, *J* = 9.6 Hz, thiophene-H*_C_*_5_), 9.90 (s, 1H, NH). ^13^C-NMR (DMSO-*d*_6_): δ = 44.20 (thiadiazine CH), 105.2 (pyridazine CH), 115.2 (CN), 120.5, 125.2, 129.9, 151.9, 152.5, 159.1, 160.7, 162.3 and 163.9. (Ar-C, C=C, C-S and C=N). Anal. Calcd. for C_12_H_8_N_8_S_2_(328.38): C, 43.89; H, 2.46; N, 34.12; S, 19.53; Found: C, 43.74; H, 2.40; N, 34.02; S, 19.44.

*N-[8-Cyano-3-(2-thienyl)-2H,8H-pyridazino[3′,4′:5,6][1,2,4]triazino[3,4-b][1,3,4]thiadiazin-9-yl]guanidine***13**. A mixture of compound **11** (0.328 g, 0.001 mol) and cyanamide (0.042 g, 0.001 mol) in ethanol (20 mL) and water (5 mL) was stirred under reflux for 1 h. The mixture then cooled and the solid formed was filtered and washed with 10 mL of ethanol, which resulted in a reddish brown precipitate that crystallized from methanol to give reddish orange crystals. Yield 65%, m.p. 278–280 °C. IR: 3354–3213 cm^−1^ (NH_2_ and 3NH), 2201 cm^−1^ (C≡N) and 1619 cm^−1^ (C=N amide). ^1^H-NMR (DMSO-*d*_6_): δ = 3.53 (s, 1H, thiadiazine CH), 5.69 (s, 1H, C=NH), 5.91 (s, 1H, pyridazine CH), 5.97 (s, 1H, C-NH-C), 6.62 (s, 2H, NH_2_), 7.03 (dd appears t, 1H, *J* = 7.2, 7.2 Hz, thiophene-H*_C_*_4_), 7.34 (d, 1H, *J* = 7.2 Hz, thiophene-H*_C_*_3_), 7.45 (d, 1H, *J* = 10.2 Hz, thiophene-H*_C_*_5_), 9.85 (s, 1H, NH). ^13^C-NMR (DMSO-*d*_6_): δ = 44.20 (S-CH-CN), 102.0 (pyridazine C), 107.4 (CN), 120.8, 125.0, 129.0, 141.3, 150.2, 151.7, 155.3, 157.2, 159.8 and 162.4 (C≡N, Ar-C, C-S and C=N). Anal. Calcd. for C_13_H_10_N_10_S_2_(370.42): C, 42.15; H, 2.72; N, 37.81; S, 17.31; Found: C, 42.03; H, 2.62; N, 37.74; S, 17.22. LCMS (ESI) *m*/*z* (int. %) (371 (4.58)) [M + H]^+^; Calcd. For (370.42): 370 (15.01), 304 (1.29), 300 (5.57), 239 (35.03), 230 (100), 202 (20.51), 168 (14.41), 134 (12.37), 134 (15.99), 108 (64.65), 98 (63.21), 82 (23.74), 69 (42.40), 68 (15.00), 63 (14.24).

*8,10-Diamino-3-(2-thienyl)-2H,7aH-pyridazino[3′,4′:5,6][1,2,4]triazino[3,4-b]pyrimido[4,5-e][1,3,4]thiadiazine***14**. A mixture of **12** (0.328 g, 0.001 mol) and thiourea and/or urea (0.001 mol) was refluxed in AcOH glacial (20 mL) containing a catalytic amount of HCl (1 mL) for 4 h. The mixture left to cool was then poured onto ice-cold water and the pH adjusted to 7 with ammonia solution. The solid formed was filtered and crystallized from ethanol to give orange-red crystals. Yield, 66% for urea and 84% for thiourea, m.p.: over 300 °C. IR: 3913–3261 cm^−1^ (2NH_2_ and NH) and 1622 cm^−1^ (C=N). ^1^H-NMR (DMSO-*d*_6_): δ = 5.52 (s, 2H, NH_2_), 6.59 (s, 2H, NH_2_), 7.03 (dd appears t, 1H, *J* = 7.2, 7.2 Hz, thiophene-H*_C_*_4_), 7.31 (d, 1H, *J* = 7.2 Hz, thiophene-H*_C_*_3_), 7.41 (d, 1H, *J* = 10.2 Hz, thiophene-H*_C_*_5_), 9.94 (s, 1H, NH). ^13^C-NMR (DMSO-*d*_6_): δ = 45.20 (S-CH-CN), 103.0 (pyridazine C), 117.9, 120.6, 121.7, 125.7, 133.1, 137.4, 147.9, 151.0, 152.4, 158.8 and 161.8 (Ar-C, C-S and C=N). Anal. Calcd. for C_13_H_10_N_10_S _2_(370.42): C, 42.15; H, 2.72; N, 37.81; S, 17.31; Found: C, 42.08; H, 2.68; N, 37.71; S, 17.30.

*[7,7-Diamino-3-(2-thienyl)-7,8-dihydro-2H-pyridazino[3,4-e][1,2,4]triazino[6,1-c]-[1,2,4]triazin-9-yl](thioxo)acetonitrile***15**. A mixture of compound **12** (0.328 g, 0.001 mol) and cyanamide (0.042 g, 0.001 mol) in ethanol (20 mL) and water (5 mL) was stirred under reflux for 4 h. The mixture then cooled and the solid formed was filtered and washed with 10 mL of ethanol, which resulted in a brownish precipitate that crystallized from methanol to give reddish brown crystals. Yield 42%, m.p. 298–300 °C. IR: 3376–3212 cm^−1^ (2NH_2_ and 2NH), 2207 cm^−1^ (C≡N), 1619 cm^−1^ (C=N amide) and 1345 cm^−1^ (C=S amide). ^1^H-NMR (DMSO-*d*_6_): δ = 3.53 (s, 1H, thiadiazine CH), 5.91 (s, 1H, pyridazine CH), 6.62 (s, 4H, 2NH_2_), 7.03 (dd appears t, 1H, *J* = 7.8, 7.2 Hz, thiophene-H*_C_*_4_), 7.33 (d, 1H, *J* = 7.2 Hz, thiophene-H*_C_*_3_), 7.45 (d, 1H, *J* = 9.6 Hz, thiophene-H*_C_*_5_), 9.91 (s, 1H, NH), 10.63 (s, 1H, NH). ^13^C-NMR (DMSO-*d*_6_): δ = 101.5 (pyridazine C), 106.1 (CN), 118.3, 120.5, 125.5, 129.9, 147.2, 151.7, 152.4, 155.0, 162.2, 165.8 and 172.4 (C≡N, Ar-C, C-S, C=S and C=N). Anal. Calcd. for C_13_H_10_N_10_S_2_ (370.42): C, 42.15; H, 2.72; N, 37.81; S, 17.31; Found: C, 42.07; H, 2.68; N, 37.77; S, 17.30.

*[8-Cyano-3-(2-thienyl)-2H,8H-pyridazino[3′,4′:5,6][1,2,4]triazino[3,4-b][1,3,4]thiadiazin-9-yl]carbamodithioic acid***16**. A mixture of compound **12** (0.328 g, 0.001 mol), carbon disulfide (1.0 mL, excess) and KOH (0.06 g, 0.01 mol) in ethanol(25mL)was heated under reflux 4 h. After cooling, the solution was poured onto ice water then acidified with dilute HCl. The red precipitate obtained was filtered, dried, and crystallized from ethanol to give red crystals. Yield, 73%, m.p.: over 300 °C. IR: 3287–3254 cm^−1^ (2NH), 2613 cm^−1^ (SH), 2201 cm^−1^ (CN), 1622 cm^−1^ (C=N) and 1345 cm^−1^ (C=S). ^1^H-NMR (DMSO-*d*_6_): δ = 3.58 (s, 1H, thiadiazine CH), 5.67 (s, 1H, pyridazine CH),7.03 (dd appears t, 1H, *J* = 7.8, 7.2 Hz, thiophene-H*_C_*_4_), 7.31 (d, 1H, *J* = 7.2 Hz, thiophene-H*_C_*_3_), 7.41 (d, 1H, *J* = 9.6 Hz, thiophene-H*_C_*_5_), 9.91 (s, 1H, NH), 10.03 (s, 1H, NH), 13.78 (s, 1H, SH).^13^C-NMR (DMSO-*d*_6_): δ = 47.20 (S-CH-CN), 101.5 (pyridazine C), 108.1 (CN), 120.5, 125.1, 129.9, 150.2, 152.7, 157.4, 159.0, 164.3, 165.1 and 174.1 (C≡N, Ar-C, C-S, C=S and C=N). Anal. Calcd. for C_13_H_8_N_8_S_4_ (404.52): C, 38.60; H, 1.99; N, 27.70; S, 31.71; Found: C, 38.44; H, 1.91; N, 27.56; S, 31.60.

*3-(2-Thienyl)-2H,7aH-pyridazino[3′,4′:5,6][1,2,4]triazino[3,4-b]pyrimido[4,5-e][1,3,4]thiadiazine-8,10(9H,11H)dithione***17**. Method A: A solution of compound **16** (0.404 g, 0.001 mol) in 25 mL ethanolic sodium ethoxide (0.07 g, 0.001 mol) was refluxed for 10 h. After cooling, the solution was poured onto ice-water and acidified with HCl. The orange solid formed was filtered and then crystallized from DMF to give compound **17**. Yield, 75%, m.p.: over 300 °C. Method B: Compound **12** (0.328 g, 0.001 mol) and CS_2_ (2 mL) in dry pyridine (10 mL) was refluxed for 13 h. After cooling, the solution was poured onto ice-water and then acidified with HCl. The obtained solid product was collected by filtration and then crystallized from DMF to give **17**. Yield 61%, m.p. over 300 °C. IR: 3312–3247 cm^1^ (3NH), 1623 cm^−1^ (C=N) and 1351–1321 cm^−1^ (2C=S). ^1^H-NMR (DMSO-*d*_6_): 4.01 (s, 1H, thiadiazine CH), 5.68 (s, 1H, pyridazine CH),7.05 (dd appears t, 1H, *J* = 7.8, 7.2 Hz, thiophene-H*_C_*_4_), 7.35 (d, 1H, *J* = 7.2 Hz, thiophene-H*_C_*_3_), 7.44 (d, 1H, *J* = 9.6 Hz, thiophene-H*_C_*_5_), 9.88 (s, 1H, NH), 10.78 (s, 1H, NH), 11.08 (s, 1H, NH). ^13^C-NMR (DMSO-*d*_6_): δ = 47.20 (S-CH-CN), 100.1 (pyridazine C), 108.1 (CN), 120.5, 125.1, 129.9, 150.2, 152.7, 163.4, 165.0, 166.2, 172.1 and 174.1 (C≡N, Ar-C, C-S, C=S and C=N). Anal. Calcd. for C_13_H_8_N_8_S_4_(404.52): C, 38.60; H, 1.99; N, 27.70; S, 31.71; Found: C, 38.51; H, 1.93; N, 27.60; S, 31.66.

*8-Amino-3-(2-thienyl)-2H,7aH-pyridazino[3′,4′:5,6][1,2,4]triazino[3,4-b]pyrimido[4,5-e][1,3,4]thiadiazine***18**. Compound **12** (0.328 g, 0.001 mol) in formamide (2 mL) and DMF (12 mL) was refluxed for 3 h. After cooling, reddish brown solid formed. This solid was collected by filtration and crystallized from ethanol. Yield, 45%, m.p.: over 300 °C. IR: 3368–3271 cm^−1^ (NH_2_ and NH), 1624 cm^−1^ (C=N). ^1^H-NMR (DMSO-*d*_6_): δ = 3.33 (s, 1H, thiadiazine CH), 5.62 (s, 1H, pyridazine CH), 6.52 (s, 2H, NH_2_), 7.03 (dd appears t, 1H, *J* = 7.2, 7.2 Hz, thiophene-H*_C_*_4_), 7.31 (d, 1H, *J* = 7.2 Hz, thiophene-H*_C_*_3_), 7.41 (d, 1H, *J* = 10.2 Hz, thiophene-H*_C_*_5_), 9.94 (s, 1H, NH).^13^C-NMR (DMSO-*d*_6_): δ = 40.20 (S-CH-CN), 101.0 (pyridazine C), 119.9, 120.0, 121.7, 125.7, 136.1, 151.9, 152.1, 156.3, 158.8, 160.2 and 161.8 (Ar-C, C-S and C=N). Anal. Calcd. for C_13_H_9_N_9_S_2_(355.40): C, 43.93; H, 2.55; N, 35.47; S, 18.04; Found: C, 43.77; H, 2.45; N, 35.41; S, 17.91.

*Ethyl [8-cyano-3-(2-thienyl)-2H,8H-pyridazino[3′,4′:5,6][1,2,4]triazino[3,4-b][1,3,4]thiadiazin-9-yl]imidoformate***19**. A mixture of compound **12** (0.328 g, 0.001 mol) and triethyl orthoformate (3 mL) in DMF (10 mL) was refluxed for 2 h. After cooling the mixture poured onto ice-water (40 mL), the precipitate formed was filtered and crystallized from ethanol to give **19** as a pale yellow powder. Yield, (54%), m.p.: over 281–283 °C. IR: 3261 cm^−1^ (NH), 2861–2850 cm^−1^ (CH_aliphatic_), 2200 cm^−1^ (CN), 1625 cm^−1^ (C=N). ^1^H-NMR (DMSO-*d*_6_): δ = 1.20 (t, 3H, *J* = 4.2, CH_3_), 3.33 (q, 2H, *J* = 3.6, CH_2_), 3.41 (s, 1H, thiadiazine CH), 5.64 (s, 1H, pyridazine CH), 7.05 (dd appears t, 1H, *J* = 7.2, 7.2 Hz, thiophene-H*_C_*_4_), 7.33 (d, 1H, *J* = 7.2 Hz, thiophene-H*_C_*_3_), 7.41 (d, 1H, *J* = 10.2 Hz, thiophene-H*_C_*_5_), 9.94 (s, 1H, NH). ^13^C-NMR (DMSO-*d*_6_): δ = 13.7 (CH_3_), 40.20 (S-CH-CN), 62.59 (CH_2_), 102.2 (pyridazine C), 116.7, 120.0, 125.7, 129.3, 150.2, 151.9, 152.1, 156.3, 158.0, 160.8 and 161.7 (CN, Ar-C, C-S and C=N). Anal. Calcd. for C_15_H_12_N_8_OS_2_ (384.44): C, 46.86; H, 3.15; N, 29.15; S, 16.68; Found: C, 46.76; H, 3.03; N, 29.00; S, 16.49.

*9-Amino-8-imino-3-(2-thienyl)-7a,8-dihydro-2H,9H-pyridazino[3′,4′:5,6][1,2,4]-triazino[3,4-b]pyrimido[4,5-e][1,3,4]thiadiazine***20**. A mixture of compound **19** (0.384 g, 0.001 mol) and hydrazine hydrate (4 mL (excess), 80%) in dimethylformamide (10 mL) was refluxed for 4h.Ayellow precipitate formed after cooling, which was filtered and crystallized from dimethylformamide. Yield, 71%, m.p.: over 300 °C. IR: 3381–3215 cm^−1^ (NH_2_, 2NH) and 1621 cm^−1^ (C=N). ^1^H-NMR (DMSO-*d*_6_): δ = 3.30 (s, 1H, thiadiazine CH), 5.52 (s, 1H, pyridazine CH), 6.14 (s, 2H, NH_2_), 7.03 (dd appears t, 1H, *J* = 7.2, 7.2 Hz, thiophene-H*_C_*_4_), 7.36 (d, 1H, *J* = 7.2 Hz, thiophene-H*_C_*_3_), 7.45 (d, 1H, *J* = 10.2 Hz, thiophene-H*_C_*_5_), 9.94 (s, 1H, NH), 10.96 (s, 1H, NH). ^13^C-NMR (DMSO-*d*_6_): δ = 45.61 (S-CH-CN), 100.8 (pyridazine C), 120.5, 125.7, 129.9, 144.5, 147.2, 152.5, 155.1, 157.3, 159.8, 162.2 and 163.1 (Ar-C, C-S and C=N). Anal. Calcd. for C_13_H_10_N_10_S_2_ (370.41): C, 42.15; H, 2.72; N, 37.81; S, 17.91; Found: C, 42.04; H, 2.61; N, 37.66; S, 17.73.

*8,10-Diamino-3-(2-thienyl)-2H,7aH-pyridazino[3′,4′:5,6][1,2,4]triazino[3,4-b]pyrido-[2,3-e][1,3,4]thiadiazine-9-carbonitrile***21**. A suspension of compound **12** (0.328 g, 0.001 mol) in ethanol (25 mL) malononitrile (0.06 g, 0.001 mol) was combined with a catalytic amount of triethylamine (0.5 mL). The mixture was refluxed for 8 h. After cooling, reddish precipitate formed, which was filtered and crystallized from dioxane to give **21.** Yield, 58%, m.p.: over 300 °C. IR: 3382–3261 cm^−1^ (2NH_2_ and NH), 2200 cm^−1^ (CN), 1618 cm^−1^ (C=N). ^1^H-NMR (DMSO-*d*_6_): δ = 3.63 (s, 1H, thiadiazine CH), 5.88 (s, 1H, pyridazine CH), 6.02 (s, 1H, NH_2_), 6.59 (s, 1H, NH_2_), 7.05 (dd appears t, 1H, *J* = 7.8, 7.2 Hz, thiophene-H*_C_*_4_), 7.33 (d, 1H, *J* = 6.6 Hz, thiophene-H*_C_*_3_), 7.43 (d, 1H, *J* = 9.6 Hz, thiophene-H*_C_*_5_), 9.90 (s, 1H, NH). ^13^C-NMR (DMSO-*d*_6_): δ = 37.91 (thiadiazine CH), 89.33 (pyridazine CH), 91.30, 116.2 (CN), 120.5, 125.2, 129.9, 141.3, 143.2, 151.1, 152.1, 159.6, 160.7, 161.3 and 163.9. (CN, Ar-C, C=C, C-S and C=N). Anal. Calcd. for C_15_H_10_N_10_S_2_ (394.44): C, 45.68; H, 2.56; N, 35.51; S, 16.26; Found: C, 45.54; H, 2.50; N, 35.42; S, 16.18.

*8-Amino-10-oxo-3-(2-thienyl)-10,11-dihydro-2H,7aH-pyridazino[3′,4′:5,6][1,2,4]triazino[3,4-b]pyrido[2,3-e][1,3,4]thiadiazine-9-carbonitrile***22**. A mixture of compound **12** (0.328 g, 0.001 mol), ethyl cyanoacetate (0.113 g, 0.001 mol) with a catalytic amount of Et_3_N in ethanol (20 mL) was refluxed for 9 h. The resulting solid mass was filtered and crystallized from dioxane to yield compound **22** as an orange powder. Yield 61%, m.p. over 300 °C. IR: 3382–3200 cm^−1^ (NH_2_ and 2NH), 2199 cm^−1^ (CN), 1659 cm^−1^ (C=O amide) and 1624 cm^−1^ (C=N). ^1^H-NMR (DMSO-*d*_6_): δ = 3.61 (s, 1H, thiadiazine CH), 5.80 (s, 1H, pyridazine CH), 6.62 (s, 1H, NH_2_), 7.05 (dd appears t, 1H, *J* = 7.8, 7.2 Hz, thiophene-H_*C*4_), 7.31 (d, 1H, *J* = 6.6 Hz, thiophene-H_*C*3_), 7.45 (d, 1H, *J* = 9.6 Hz, thiophene-H_*C*5_), 9.90 (s, 1H, NH), 11.43 (s, 1H, NH). ^13^C-NMR (DMSO-*d*_6_): δ = 39.84 (thiadiazine CH), 89.03 (pyridazine CH), 94.30, 116.2 (CN), 120.5, 125.2, 129.9, 148.3, 149.2, 151.1, 153.2, 158.7, 160.7, 162.3 and 164.9. (CN, Ar-C, C=C, C-S, C=N and C=O). Anal. Calcd. for C_15_H_9_N_9_OS_2_ (395.42): C, 45.56; H, 2.29; N, 31.88; S, 16.22; Found: C, 45.38; H, 2.18; N, 31.71; S, 16.11.

*[8-Oxo-3-(2-thienyl)-8,9-dihydro-2H,7aH-pyridazino[3′,4′:5,6][1,2,4]triazino[3,4-b]pyrimido[4,5-e][1,3,4]thiadiazin-10-yl]acetonitrile***23**. Compound **12** (0.328 g, 0.001 mol) with ethyl cyanoacetate (10 mL) was refluxed for 5 h. The solid formed was filtered and then crystallized from dioxane to give compound **23**. Yield 59%, m.p. over 300 °C. IR: 3259–3221 cm^−1^ (2NH), 2200 cm^−1^ (CN), 1668 cm^−1^ (C=O amide) and 1623 cm^−1^ (C=N). ^1^H-NMR (DMSO-*d*_6_): δ = 3.23 (s, 2H, CH_2_CN), 3.66 (s, 1H, thiadiazine CH), 5.67 (s, 1H, pyridazine CH), 7.04 (dd appears t, 1H, *J* = 7.8, 7.2 Hz, thiophene-H_*C*4_), 7.34 (d, 1H, *J* = 6.6 Hz, thiophene-H_*C*3_), 7.40 (d, 1H, *J* = 9.6 Hz, thiophene-H_*C*5_), 9.82 (s, 1H, NH), 10.56 (s, 1H, NH). ^13^C-NMR (DMSO-*d*_6_): δ = 22.83 (CH_2_), 45.23 (thiadiazine CH), 84.03 (pyridazine CH), 120.5 (CN), 122.0, 125.2, 129.4, 149.2, 152.1, 153.2, 157.2, 161.7, 163.3, 164.3 and 166.1. (CN, Ar-C, C=C, C-S, C=N and C=O). Anal. Calcd. for C_15_H_9_N_9_OS_2_ (395.42): C, 45.56; H, 2.29; N, 31.88; S, 16.22; Found: C, 45.41; H, 2.23; N, 31.74; S, 16.06.

*N-[8-Cyano-3-(2-thienyl)-2H,8H-pyridazino[3′,4′:5,6][1,2,4]triazino[3,4-b][1,3,4]thiadiazin-9-yl]acetamide***24**. A solution of compound **12** (0.328 g, 0.001 mol) in acetic anhydride (10 mL) was refluxed for 6 h. The reaction mixture was poured onto ice-water and the resulting solid was filtered and crystallized from ethanol to give **24** as yellow crystals. Yield 69%, m.p. 284–286 °C. IR: 3244–3232 cm^−1^ (2NH), 2199 cm^−1^ (CN), 1660 cm^−1^ (C=O amide) and 1622 cm^−1^ (C=N). ^1^H-NMR (DMSO-*d*_6_): δ = 1.99 (s, 3H, CH_3_), 3.65 (s, 1H, thiadiazine CH), 5.71 (s, 1H, pyridazine CH), 7.04 (dd appears t, 1H, *J* = 7.8, 7.2 Hz, thiophene-H*_C_*_4_), 7.33 (d, 1H, *J* = 6.6 Hz, thiophene-H*_C_*_3_), 7.42 (d, 1H, *J* = 9.6 Hz, thiophene-H*_C_*_5_), 9.91 (s, 1H, NH), 11.56 (s, 1H, NH). ^13^C-NMR (DMSO-*d*_6_): δ = 25.23 (CH_2_), 41.30 (thiadiazine CH), 91.27 (pyridazine CH), 114.6, 120.5 (CN), 125.1, 129.5, 137.2, 144.5, 149.1, 150.1, 151.2, 154.2 and 162.1. (CN, Ar-C, C=C, C-S, C=N and C=O). Anal. Calcd. for C_14_H_10_N_8_OS_2_(370.41): C, 45.40; H, 2.72; N, 30.25; S, 17.31; Found: C, 45.28; H, 2.65; N, 30.21; S, 17.20.

### 3.2. Anti-Tumor Activity Assay

#### Methods

The tested human carcinoma cell lines were obtained from the American Type Culture Collection (ATCC, Rockville, MD, USA). The cells were grown on RPMI-1640 medium supplemented with 10% heat-inactivated fetal calf serum, 1% l-glutamine, and 50 µg/mL gentamycin at 37 °C in a humidified atmosphere with a 5% CO_2_ incubator (Shel lab 2406, Candler, NC, USA).

For anti-tumor assays, the tumor cell lines were suspended in medium at concentration 5 × 10^4^ cell/well in Corning^®^ 96-well tissue culture plates and then incubated for 24 h. The tested compounds were then added into 96-well plates (three replicates) to achieve ten concentrations for each compound (started from 500 to 1 µg/mL). Six vehicle controls with media or 0.1% DMSO were run for each 96-well plate as a control. After incubating for 24 h, the numbers of viable cells were determined by the MTT assay [33]. Briefly, the media was removed from the 96-well plate and replaced with 100 µL of fresh culture RPMI 1640 medium without phenol red. Then, 10 µL of the 12 mM MTT (3-[4,5-dimethylthiazol-2-yl]-2,5-diphenyltetrazolium bromide (Sigma, Taufkirchen, Germany) was added to each well including the untreated controls. The 96-well plates were then incubated at 37 °C and 5% CO_2_ for 4 h. An 85 µL aliquot of the media was removed from the wells and 50 µL of DMSO was added to each well and mixed thoroughly with the pipette and incubated at 37 °C for 10 min. Then, the optical density was measured at 590 nm with the microplate reader (SunRise, TECAN, Mannedorf, Switzerland)) to determine the number of viable cells and the percentage of viability was calculated as [(ODt/ODc)] × 100% where ODt is the mean optical density of wells treated with the tested sample and ODc is the mean optical density of untreated cells. The relation between surviving cells and drug concentration is plotted to obtain the survival curve of each tumor cell line after treatment with the specified compound. The 50% inhibitory concentration (IC_50_), which is the concentration required to cause toxic effects in 50% of intact cells, was estimated from graphic plots of the dose–response curve for each concentration using Graphpad Prism software (San Diego, CA, USA) [34].

## 4. Conclusions

Novel derivatives of fused 1,2,4-triazines were obtained via subsequent reaction methodology. The new synthesized compounds were screened against three cell lines, HepG2, HCT-116 and MCF-7, to discover their anti-cancer activity. The results revealed that all the tested compounds showed inhibitory activity against the tumor cell lines in a concentration-dependent manner. We plan to evaluate the affinity and selectivity of these and other related synthetic compounds towards adenosine receptor subtypes A_2A_ and A_3_. The results will be used to optimize these structures for biological activity and our conclusions will be reported in a future publication.

## Figures and Tables

**Table 1:** The anti-tumor activities of the tested compounds expressed as IC_50_ values and compared with reference standard drugs evaluated on breast cancer and liver cancer cell lines.

Sample Code	MCF-7	HepG-2	HCT-116
2	29.6 ± 0.9	25 ± 0.4	30.9 ± 0.4
12	43.9 ± 1.2	28.6 ± 0.9	34.9 ± 0.6
3	54 ± 0.8	35.5 ± 1.2	46.2 ± 0.7
4	54.5 ± 1.2	30.2 ± 0.4	45.4 ± 0.8
5	168 ± 4.5	102 ± 0.9	160 ± 2.3
6	100 ± 0.9	30.7 ± 0.7	59.3 ± 0.8
7	78.2 ± 1.2	35.6 ± 0.9	59.5 ± 1.2
10	404 ± 3.4	206 ± 2.3	244 ± 3.9
16	29.2 ± 0.7	23.6 ± 0.6	27.4 ± 0.6
Cisplatin	5.71 ± 0.4	3.67 ± 0.2	2.43 ± 0.2
